# Current Non-operative Management of Shoulder Pathologies: A Narrative Review

**DOI:** 10.7759/cureus.98500

**Published:** 2025-12-05

**Authors:** Ricardo A Vargas-Figueroa, Derick Rodríguez-Reyes, Hiram E Luigi Martinez, Amanda S Vazquez-Lloret, Rafael Señeriz Ortiz

**Affiliations:** 1 Department of Orthopaedic Surgery, Ponce Health Sciences University, Ponce, PRI; 2 Orthopaedic Surgery, Universidad Central del Caribe, Bayamón, PRI

**Keywords:** acromioclavicular joint, biceps tendinopathy, glenohumeral osteoarthritis, idiopathic adhesive capsulitis, physical therapy modalities, rotator cuff pathology, rotator cuff tears, selective non-operative management, shoulder instability, slap tear

## Abstract

Shoulder disorders, including rotator cuff-related pain, adhesive capsulitis, impingement, glenohumeral osteoarthritis (GHOA), acromioclavicular (AC) pathology, instability, and biceps/superior labrum anterior to posterior (SLAP) lesions, are prevalent causes of pain, disability, and healthcare utilization. Contemporary care emphasizes non-operative strategies as first-line management, with surgery reserved for refractory symptoms or specific structural indications. The researcher conducted a systematized narrative review of adult non-operative management for common shoulder pathologies. Targeted searches of Medical Literature Analysis and Retrieval System Online (MEDLINE) (via PubMed), the Cochrane Library, and guideline repositories (American Academy of Orthopaedic Surgeons (AAOS)/American Shoulder and Elbow Surgeons (ASES)/American Physical Therapy Association (APTA)/American College of Rheumatology (ACR)) were performed from 2010 to October 2025. The researcher prioritized clinical practice guidelines, Cochrane and systematic reviews (including network meta-analyses), and randomized trials. Studies focused on non-operative interventions with patient-centered outcomes were qualitatively synthesized by pathology; no quantitative pooling was performed. Exercise-based rehabilitation is the cornerstone across conditions, producing clinically meaningful pain and functional gains and, in subacromial pain, outcomes comparable to surgery. For adhesive capsulitis, intra-articular corticosteroid, hydrodilatation, and suprascapular nerve block yield short-term benefits that are greatest when paired with mobilization and stretching. In GHOA, individualized range of motion (ROM) maintenance and strengthening, activity adaptation, and time-limited injections (corticosteroid; hyaluronic acid with variable evidence) dominate care; platelet-rich plasma remains investigational. For instability, early ROM followed by rotator cuff/scapular strengthening and proprioceptive training is standard, with bracing for select athletes. Acute low-grade AC injuries respond to brief sling use.

## Introduction and background

Shoulder disorders, including rotator cuff-related shoulder pain, adhesive capsulitis, impingement syndrome, glenohumeral osteoarthritis (GHOA), acromioclavicular (AC) joint pathology, shoulder instability, and biceps/superior labrum anterior to posterior (SLAP) lesions, are among the most common musculoskeletal complaints in adult practice. Each condition represents a distinct clinical entity characterized by pain, restricted range of motion, or functional impairment, and diagnostic clarity is essential for appropriate non-operative management [1,2]. Lifetime prevalence of shoulder pain approaches 70%, with monthly prevalence estimates of 18-31% in the general population; the burden rises with age and is associated with disability, sleep disruption, productivity loss, and substantial healthcare utilization [1,2].

First-line care for most atraumatic and degenerative conditions is non-operative, typically a combination of education, activity modification, exercise-based physical therapy, and judicious use of pharmacologic and image-guided interventions, with surgery reserved for refractory cases or discrete structural indications [3-6]. The novelty of this review lies in consolidating contemporary evidence and guideline-informed recommendations across all major shoulder pathologies into a single, clinically focused synthesis to support everyday orthopaedic and sports medicine decision-making.

## Review

Methods

Design and Scope

This review conducted a systematized narrative synthesis focused on adult shoulder pathologies managed non-operatively. Because the goal was to inform clinical practice without quantitative pooling, the review prioritized recent clinical practice guidelines, Cochrane and systematic reviews, meta-analyses (including network meta-analyses), and pragmatic randomized controlled trials (RCTs).

Sources and Search

Targeted searches of MEDLINE (via PubMed), the Cochrane Library, and guideline repositories for the American Academy of Orthopaedic Surgeons (AAOS), American Shoulder and Elbow Surgeons (ASES), American Physical Therapy Association (APTA), and American College of Rheumatology (ACR) (2010-October 2025) were performed, supplemented by a semantic literature scan to ensure coverage of recent syntheses and umbrella reviews. Search terms combined condition keywords (e.g., "rotator cuff," "adhesive capsulitis," "glenohumeral osteoarthritis," "shoulder instability," "acromioclavicular," and "SLAP") with intervention-related terms (e.g., "exercise," "physical therapy," "manual therapy," "NSAIDs," "corticosteroid injection," "platelet-rich plasma," "hyaluronic acid," "hydrodilatation," "shockwave," and "nerve block").

Eligibility

Human studies evaluating non-operative interventions with patient-centered outcomes (pain, function, range of motion, return to activity/activities of daily living (ADLs)) were prioritized, including RCTs, systematic reviews, Cochrane reviews, and high-quality observational studies. Studies focused primarily on operative interventions, postoperative rehabilitation, case reports (<5 patients), or non-peer-reviewed material were excluded.

Study Selection and Synthesis

Titles and abstracts were screened to identify high-level evidence per pathology. Data were summarized qualitatively by pathology and intervention domain (exercise/rehabilitation, pharmacologic, injections, and adjunct modalities). No meta-analysis or formal Grading of Recommendations, Assessment, Development, and Evaluation (GRADE) assessment was performed; limitations and evidence gaps are addressed in the Discussion. Exercise interventions across included studies followed FITT (frequency, intensity, time, type)-consistent principles, although specific parameters varied by pathology and study design. Summary recommendations are presented in Table 1, and injection-specific considerations are presented in Table 2.

**Table 1:** Recommended first-line non-operative strategies by pathology AC, acromioclavicular; SLAP, superior labrum anterior to posterior; ER, external rotation; GH, glenohumeral; HA, hyaluronic acid; PRP, platelet-rich plasma; RC, rotator cuff; UGN, ultrasound-guided needling; US, ultrasound; ROM, range of motion; NSAIDs, nonsteroidal anti-inflammatory drugs

Pathology	First-line interventions	Adjuncts/injections	Representative evidence
Rotator cuff-related shoulder pain	Education; activity modification; exercise emphasizing scapular control and progressive RC/periscapular strengthening	Short-term subacromial corticosteroid to facilitate rehab; PRP in selected chronic tendinopathy; ESWT/UGN for calcific disease	[7-15,19,23,26-33]
Adhesive capsulitis	Supervised mobilization + home exercise; stage-specific education and analgesia	Intra-articular corticosteroid (early), hydrodilatation for ROM/pain, suprascapular nerve block for refractory pain	[4,35,40-49]
Glenohumeral osteoarthritis	Activity adaptation; ROM maintenance; periarticular strengthening	Intra-articular corticosteroid; consider HA (variable evidence); PRP investigational	[50-55,62-64]
Shoulder instability	Brief immobilization for comfort; early ROM; RC/scapular strengthening; proprioceptive training	Selective bracing in contact athletes; surgical referral for high-risk features or failed rehab	[65-71,75-77]
AC joint pathology	Acute: short sling + early mobilization; Chronic: activity modification, targeted therapy for scapular mechanics	US-guided corticosteroid for recalcitrant AC arthritis	[78-87]
Biceps/SLAP	Avoid repetitive overhead activity; eccentric biceps + scapular stabilization; NSAIDs/analgesics	US-guided corticosteroid into bicipital groove (short-term) in select cases	[88-99]

**Table 2:** Injection-based therapies: expected benefit window and cautions PRP, platelet-rich plasma; GH, glenohumeral; GHOA, glenohumeral osteoarthritis; OA, osteoarthritis; ER, external rotation

Modality	Primary indications	Typical benefit window	Key cautions/notes	Representative evidence
Subacromial corticosteroid	Rotator cuff-related shoulder pain impairing rehab participation	Weeks (up to ~8-12); diminishing thereafter	Avoid repeated dosing; potential tendon effects; use to facilitate exercise	[15-21]
PRP	Chronic rotator cuff tendinopathy or partial tears; select adhesive capsulitis cases	Medium term (3-12 months) in some trials; heterogeneous effects	Formulation/technique variability; cost/access; adjunct to structured rehab	[16-25,116-120]
Intra-articular corticosteroid (GH)	Symptomatic flares of GHOA	Weeks to a few months	Transient relief only; consider joint health with repeats	[50,53,62]
Hyaluronic acid (GH)	GHOA refractory to first-line care	Months in some studies	Evidence quality lower vs. knee OA; variable response	[53,63]
Hydrodilatation + steroid	Adhesive capsulitis with marked ER restriction	Up to six months	Pair with mobilization/therapy; image guidance recommended	[45,47-48]
Suprascapular nerve block	Adhesive capsulitis with refractory pain or steroid contraindications	Short to medium term	Adjunct to therapy; image guidance improves accuracy	[42,46,48, 49]

Current non-operative management options

Rotator Cuff-Related Shoulder Pain (Tendinopathy, Partial Tears, Impingement)

Education and activity modification: Advise avoidance of provocative overhead activity during symptomatic phases; graduated reintroduction occurs as pain and function improve [3,7,8].

Exercise-based care: Structured rehabilitation emphasizing scapular stabilization, motor control, and progressive rotator cuff/periscapular strengthening is the cornerstone of care, with consistent reductions in pain and improvements in function within approximately 12 weeks in prospective cohorts and RCT-informed programs [7-13]. Manual therapy can add small, short-term benefits when combined with exercise [8].

Pharmacologic therapy: Oral or topical nonsteroidal anti-inflammatory drugs (NSAIDs) yield modest short-term analgesia; risks require gastrointestinal (GI)/renal/cardiovascular (CV) risk stratification [3,14].

Injections: Subacromial corticosteroid injection provides short-term relief (weeks) that can facilitate rehabilitation, but benefits wane, and repeated dosing may impair tendon biology [15-21]. Platelet-rich plasma (PRP) shows mixed but emerging evidence for medium-term improvement versus corticosteroid in some trials and meta-analyses; effects are modest and heterogeneous, and PRP should be considered adjunctive and patient selective [16-25].

Adjuncts for calcific tendinopathy: High-energy extracorporeal shockwave therapy (ESWT) and ultrasound-guided needling (often with subacromial steroid) are effective options in persistent calcific disease; ESWT benefits are less consistent in non-calcific tendinopathy [26-33].

Adhesive Capsulitis (Frozen Shoulder)

Natural history and education: Typically self-limited but prolonged across freezing-frozen-thawing phases; a subset may have lasting limitations [34-36]. Analgesics (NSAIDs/acetaminophen) may help early pain without altering the course [37-39].

Rehabilitation and mobilization: Supervised mobilization and stretching, combined with home exercise, improve pain and function; manual therapy can augment gains, particularly for external rotation [4,40-44].

Image-guided therapies: Intra-articular corticosteroid injection, especially early, improves short-term pain and function and is most effective when paired with therapy [35,40,42,44-47]. Hydrodilatation (capsular distension) offers a superior short-term range of motion (ROM)/pain gains versus steroid alone in several analyses [45,47,48]. Suprascapular nerve block is a reasonable adjunct for refractory pain or steroid contraindications [42,46,48,49].

Glenohumeral Osteoarthritis

Lifestyle and rehabilitation: Activity adaptation plus individualized therapy to maintain ROM and strengthen periarticular musculature form the foundation of care and can delay arthroplasty in selected patients [50-55].

Pharmacologic therapy: NSAIDs are first-line for symptomatic control (extrapolated largely from knee/hip OA); acetaminophen has limited efficacy; duloxetine can help centrally mediated pain or NSAID-intolerant patients [53,56-61].

Injections: Intra-articular corticosteroid affords temporary relief; HA may benefit some patients, though evidence quality is lower than in knee OA; PRP remains investigational with early supportive data but no consensus for routine use [50,53,62-64].

Shoulder Instability (Recurrent Subluxation/Dislocation)

Acute care and rehabilitation: Prompt reduction, brief immobilization for comfort, early ROM, then progressive rotator cuff and scapular strengthening and proprioceptive training are standard; prolonged immobilization is discouraged [65-70]. Selective in-season bracing may aid contact athletes [65,71].

Surgical referral: Consider for failure of structured rehabilitation, high-risk sport exposure, or critical bone loss or engaging lesions; risk stratification (younger age, male sex, collision sports) informs decisions [71-77].

Acromioclavicular Joint Pathology

Acute low-grade sprains: Short sling for comfort (one to two weeks), early mobilization, and NSAIDs/acetaminophen typically suffice; most return to activity within two to four weeks [78-82].

Chronic acromioclavicular arthritis: Activity modification, NSAIDs (or acetaminophen if needed), targeted therapy for scapular mechanics, and consideration of ultrasound-guided corticosteroid injection for recalcitrant pain; distal clavicle excision is reserved for failures of comprehensive conservative care [83-87].

Biceps Tendinopathy and Slap Lesions

First-line: Activity modification to limit repetitive overhead loading, combined with NSAIDs/analgesics and a criteria-based rehabilitation program emphasizing scapular control and eccentric biceps loading [88-97].

Injections: Ultrasound-guided corticosteroid into the bicipital groove may provide short-term pain relief in selected cases; avoid repeat dosing due to tendon risks [94,95,98,99].

Physical Modalities and Adjunct Therapies

Heat or ice can provide short-term symptom relief; therapeutic ultrasound and transcutaneous electrical nerve stimulation (TENS) show limited additive benefit over exercise in most contexts [33,94,100-105]. ESWT demonstrates moderate evidence for calcific tendinopathy; effects are smaller and less consistent in non-calcific disease [27,28,106]. Manual therapy and joint mobilization are useful adjuncts, especially for adhesive capsulitis and rotator cuff-related pain when combined with structured exercise [4,8,40,42,43,107-110].

Discussion

Across shoulder pathologies, exercise-based rehabilitation remains the central pillar of conservative care, supported by guidelines and high-level syntheses [3,4,8,50,111-113]. For rotator cuff-related shoulder pain and subacromial syndromes, therapeutic exercise, with or without manual therapy, achieves outcomes comparable to acromioplasty in many settings and reduces reliance on injections [8,15,19,113,114]. For adhesive capsulitis, image-guided interventions, including intra-articular corticosteroid, hydrodilatation, and suprascapular nerve block, provide clinically meaningful short-term benefits that synergize with mobilization and stretching [35,40-49,115]. In GHOA, multimodal conservative care integrating activity modification, ROM maintenance, strengthening, and time-limited injections can defer arthroplasty for selected patients [50-55,62-64].

The role of biologics, most notably PRP, continues to evolve. Meta-analytic signals suggest potential medium-term benefits for rotator cuff tendinopathy and possibly early adhesive capsulitis, but heterogeneity in preparations and protocols, modest effect sizes, and cost and access considerations temper routine adoption [16-25,116-122]. Adjunct modalities, such as ultrasound and TENS, offer at best small, short-term effects and should not replace active exercise programs [33,100-105].

Limitations include heterogeneity in diagnostic labels and rehabilitation protocols, short follow-up in many trials, potential performance and placebo effects in injection studies, and limited shoulder-specific data for several pharmacologic agents extrapolated from knee and hip osteoarthritis (OA) [27,28,33,53,56-59]. Demographic-specific comparative effects, such as age and sex, could not be evaluated without formal quantitative pooling; these represent important areas for future research. This review is narrative and non-registered, without formal risk-of-bias grading; nonetheless, the review prioritized high-level evidence to enhance clinical applicability. Future work may benefit from graphical comparative summaries or visual decision aids, though such figures were not included here to avoid implying quantitative weighting where none was performed.

## Conclusions

Most atraumatic shoulder conditions improve with a structured, patient-centered program integrating education, activity modification, and exercise-based rehabilitation. Short-term pharmacologic and image-guided therapies can accelerate early gains or enable participation. Surgery is reserved for refractory symptoms or specific structural indications, such as recurrent instability with bone loss. The synthesized evidence presented here underscores the need for standardized rehabilitation protocols and pragmatic comparative effectiveness research to guide future clinical decision-making.
